# Epoxidized Soybean-Oils-Based Pressure-Sensitive Adhesives with Di-Hydroxylated Soybean-Oils Copolymerizing and Antioxidant Grafting

**DOI:** 10.3390/polym15122709

**Published:** 2023-06-16

**Authors:** Yongyan Kuang, Wenlong Li, Shuli Xie, Weijian Gong, Zihan Ye, Yiming Wang, Dan Peng, Jun Li

**Affiliations:** 1 College of Food Science and Engineering, Henan University of Technology, Zhengzhou 450001, China; k2020920035@stu.haut.edu.cn (Y.K.); 2021920094@stu.haut.edu.cn (W.L.); 202020010210@stu.haut.edu.cn (W.G.); 202020010207@stu.haut.edu.cn (Z.Y.); 221170100201@stu.haut.edu.cn (Y.W.); 2 Ningbo Fotile Kitchen Ware Company, Ningbo 315336, China; xiesl@fotile.com

**Keywords:** pressure-sensitive adhesives, epoxidized soybean oils, di-hydroxylated soybean oils, antioxidant grafting, binding strengths, aging-resistant properties

## Abstract

Vegetable-oils-based pressure-sensitive adhesives (PSAs) are being developed as a substitute for petrochemical-based PSAs for application in daily life. However, vegetable-oils-based PSAs face the problems of unsatisfactory binding strengths and easy aging. In this work, the grafting of antioxidants (tea polyphenol palmitates, caffeic acid, ferulic acid, gallic acid, butylated hydroxytoluene, tertiary butylhydroquinone, butylated hydroxyanisole, propyl gallate (PG), tea polyphenols) was introduced into an epoxidized soybean oils (ESO)/di-hydroxylated soybean oils (DSO)-based PSA system to improve the binding strengths and aging-resistant properties. PG was screened out as the most suitable antioxidant in the ESO/DSO-based PSA system. Under optimal conditions (ESO/DSO mass ratio of 9/3, 0.8% PG, 55% rosin ester (RE), 8% phosphoric acid (PA), 50 °C, and 5 min), the peel adhesion, tack, and shear adhesion of the PG-grafted ESO/DSO-based PSA increased to 1.718 N/cm, 4.62 N, and >99 h, respectively, in comparison with the control (0.879 N/cm, 3.59 N, and 13.88 h), while peel adhesion residue reduced to 12.16% in comparison with the control (484.07%). The thermal stability of the ESO/DSO-based PSA was enhanced after PG grafting. PG, RE, PA, and DSO were partially crosslinked in the PSA system, with the rest being free in the network structures. Thus, antioxidant grafting is a feasible method for improving the binding strengths and aging-resistant properties of vegetable-oils-based PSAs.

## 1. Introduction

Petroleum-based materials have been widely applied in various industries due to their excellent performance. However, the growing concerns regarding the rapid depletion of petroleum resources and environmental issues are driving many industries to develop bio-based materials to replace petroleum-based materials partially and even completely. One of such examples is pressure-sensitive adhesives (PSAs), which are widely used in daily life in products such as tapes, labels, protective films, etc. [1,2].

Bio-based PSAs, especially vegetable-oils-based PSAs, have been considered to be a promising potential alternative to petroleum-based PSAs, which are mainly derived from acrylics, silicones, ethylene vinyl acetates, polyurethanes, polyisoprenes, polybutadienes, etc. [3,4]. Vegetable oils are mainly triglycerides in which glycerol is esterified with three saturated or unsaturated fatty acids. Polyunsaturated fatty acids in most vegetable oils except for tung oils contain methylene-interrupted 1,4-diene units (nonconjugated) rather than 1,3-diene units (conjugated) [5]. Thus, most vegetable oils are not directly polymerized for PSA preparation because of the low reactivity of the 1,4-diene unit. During the preparation of vegetable-oils-based PSA, vegetable oils are usually epoxidized initially by peroxyacids to improve the reactivity [6], followed by self-polymerizing or copolymerizing with other monomers [7,8]. The PSAs produced through self-polymerization of epoxidized vegetable oils usually have too high a degree of crosslinking but too small a number of polar functional groups because of the strong reactivity of epoxy groups, leading cohesion to be much more than adhesion and both peel and tack adhesions to be small [9]. To address these issues, most previous studies focus on the development of vegetable-oils-based PSAs through copolymerization of epoxidized vegetable oils with other monomers such as di-/polyols, acids and amines, hydroxy acids, etc. [10]. For example, Ahn et al. [11] reported that the peel adhesion reaches 1.43 N/cm for the PSA produced by copolymerization of epoxidized soybean oils (ESO) and hydroxylated soybean oils (ratio of 4:1) with phosphoric acid (10%) as the catalyst. In addition, PSA preparation using epoxidized fatty acids and their methyl esters has also been reported [11]. Like vegetable oils, however, vegetable-oils-based PSAs face easy aging issues under the actions of oxygen, heating, moisture, and ultraviolet radiation in the surroundings [12], because PSA polymer contains the residual double bonds caused by the incomplete conversion of peroxidization as well as a large number of ester bonds.

Antioxidants are mainly polyhydroxyl and/or carboxyl phenolic substances, which are widely used in food, cosmetics, medicine, and chemical industries due to their excellent oxidative-resistant, aging-resistant, and ultraviolet-resistant properties [13,14,15]. The functional hydroxyl and carboxyl groups of antioxidants possess good reactivity and, thus, can be used as co-monomers to participate in the crosslinking polymerization of PSAs. Therefore, it is expected that grafting antioxidants onto vegetable-oils-based PSAs is a great pattern for improving the binding strengths and aging-resistant properties of PSAs. However, reports of deep and comprehensive investigations into the grafting of antioxidants onto vegetable-oils-based PSAs are rare.

In this work, di-hydroxylated soybean oils (DSO) derived from the ring opening of ESO were used to copolymerize with ESO to prepare ESO/DSO-based PSAs, onto which common antioxidants were grafted synchronously. It was expected that the introduction of both DSO copolymerizing and antioxidant grafting would improve both the binding strengths and the aging-resistant properties of the resulting PSAs. The proper antioxidant was screened out initially and then grafted onto the ESO/DSO-based PSAs for optimization of preparation parameters. In addition, the thermal properties of the antioxidant-grafted ESO/DSO-based PSAs were investigated using differential scanning calorimetry (DSC) and thermogravimetric analysis (TGA), and the crosslinking mechanism of the antioxidant-grafted ESO/DSO-based PSAs was revealed by Fourier-transform infrared spectroscopy (FTIR) and nuclear magnetic resonance spectroscopy (NMR). This work could provide a feasible solution to address the easy aging issues of vegetable-oils-based PSAs, thereby improving their competitiveness in the whole PSA market.

## 2. Materials and Methods

### 2.1. Chemicals and Materials

ESO (6.12% epoxy value) was purchased from MackLin Co., Ltd. (Shanghai, China). DSO (0.91% epoxy value, hydroxyl value of 221.73 mg KOH/g) was made in the laboratory by the ring opening of ESO where α-pyrrolidone hydrogen sulfate was used as the catalyst with water as the ring-opening agent and acetone as the solvent. Rosin ester (RE, Shuanghui Chemical Co., Ltd., Wuxi, China) was used as the tackifier. An amount of 85% phosphoric acid (PA, Tianjin Kermel Chemical Reagent Co., Ltd., Tianjin, China) was used as the catalyst and crosslinker. The nine common antioxidants used were tea polyphenols (TP, purity of 98%), tea polyphenol palmitates (TPP, purity of 70%), butylated hydroxyanisole (BHA, purity of 98%), butylated hydroxytoluene (BHT, purity of 99%), tertiary butylhydroquinone (TBHQ, purity of 98%), propyl gallate (PG, purity of 98%), caffeic acid (CA, purity of 98%), ferulic acid (FA, purity of 99%), and gallic acid (GA, purity of 98.5%). The acetone and ethyl acetate used were of analytical grade. PET film (25 µm thickness) was purchased from Suzhou Dongxuan Plastic Products Co., Ltd. (Suzhou, China).

### 2.2. PSA Preparation

To 10 mL of ethyl acetate were added 10 g of ESO, designated amounts (ESO/DSO mass ratio of 9/1–9/5) of DSO, 0.4–1.2% (on the basis of ESO weight, similarly hereinafter) of antioxidant (TP, TPP, BHA, BHT, TBHQ, PG, CA, FA, and GA), and 35–75% of RE, and the mixture was vortexed until the additions were completely dissolved. The mixture was cooled in a cold bath at 0 °C for 20 min. After that, 5–9% of PA was pipetted into the mixture, immediately followed by room-temperature vortexing for 30 s and thermal (40–60 °C) sonicating for 30 s by turns. Each complete turn was counted as 1 min of reaction time, and the reaction times ranged from 3 to 7 min. After the reaction was finished, the solution was coated to 80 μm thickness on PET films, and this was followed by thermal curing at 110 °C for 30 s and then placing in a constant temperature (23 °C) and humidity (50%) chamber for 24 h before binding strength tests.

### 2.3. Aging Experiments

The dry-heat aging experiment for the PSAs was conducted with reference to ASTM D3611-06 [16], with a minor modification (humidity controlling was excluded). The PSAs were aged at 66 °C for 96 h, conditioned at room temperature for 4 h, and then tested for 180° peel adhesion. The peel adhesion residue was calculated as in Equation (1).

Peel adhesion residue% = (|peel adhesion after aging-peel adhesion before aging|)/(peel adhesion before aging) × 100(1)

### 2.4. Peel Adhesion Test

The peel adhesion of the PSAs was tested according to Method A in ASTM D3330/D3330M-04 [17]. Briefly, a 2 kg steel roller was placed onto the PSA stripes (25 mm width × 300 mm length) and then horizontally rolled back and forth three times to allow the PSA stripes to bind to the steel plate. The peel adhesion was tested with a peeling rate of 300 mm/min.

### 2.5. Tack Test

The tack of the PSAs was tested according to Method A in ASTM D6195-03 [18]. Briefly, PSA stripes (25 mm width × 175 mm length) were bent to form an annular teardrop, followed by clamping onto the upper gripper. The upper gripper moved downwards until the PSA stripes completely contacted with the center of the steel plate, and then tack was tested by switching the upper gripper to move upwards at a speed rate of 300 mm/min within 3 s.

### 2.6. Shear Adhesion Test

The shear adhesion of the PSAs was tested according to Method A in ASTM D3654/D3654M-06 [19]. Briefly, a 2 kg steel roller was placed onto the PSA stripes (25 mm width × 150 mm length) and then horizontally rolled back and forth three times to allow the PSA stripes to bind on the steel plate with a binding area of 25 mm × 25 mm. A 1 kg counterweight was hung down vertically at the free end of each PSA strip. The length of time that it took for the stripe to completely separate from the steel plate was recorded as shear adhesion.

### 2.7. Fourier-Transform Infrared Spectroscopy Measurement

The PSAs were scanned using a Fourier-transform infrared spectrometer (ALPHA, Bruker Optics Inc., Ettlingen, Germany) with PET film as the background. Scanning times were set at 32. Resolution was set at 4 cm^−1^. Spectral data were collected in the range of 4000–400 cm^−1^.

### 2.8. Differential Scanning Calorimetry Measurement

A differential scanning calorimeter (Q200, NETZSCH Group, Selb, Germany) was used to measure the DSC thermograms of the PSAs. Approximately 5 mg of PSAs scraped off from PSA strips was weighed into a hermetic pan. The measurement temperature was initially set at −60 °C and then elevated to 250 °C at 10 °C/min.

### 2.9. Thermogravimetric Measurement

A thermogravimetric analyzer (STA 449 F3/F5, NETZSCH Group, Selb, Germany) was used to measure the thermogravimetric thermograms of the PSAs. Approximately 5 mg of PSAs scraped off from PSA strips was weighed into a crucible. The measurement was under nitrogen atmosphere with temperature initially set at 20 °C and then elevated to 850 °C at 20 °C/min.

### 2.10. Hydrogen Nuclear Magnetic Resonance Spectroscopy Measurement

The ESO/DSO-based PSA (control, with RE) and the PG-grafted ESO/DSO-based PSA (without RE) were each soaked in ethyl acetate for 4 h. The PG-grafted ESO/DSO-based PSA (with RE) was soaked in anhydrous ethanol for 4 h. After that, the respective upper solutions were collected and were called “soaking solution”. Each of the undissolved adhesives was washed with its corresponding soaking solvent, followed by centrifugating at 5000 rpm for 5 min. The washing and centrifugating process was repeated 3 times. After that, the adhesive residues were dried under nitrogen protection, and called ESO/DSO-based PSA (with RE) residue after soaking, PG-grafted ESO/DSO-based PSA (without RE) residue after soaking, and PG-grafted ESO/DSO-based PSA (with RE) residue after soaking, respectively. A nuclear magnetic resonance spectrometer (AVANCE III 600M, Bruker Optics Inc., Ettlingen, Germany) equipped with a 5 mm probe was used to measure the ^1^H NMR spectra of those samples. Approximately 10 mg of each solid sample or 0.5 mL of each liquid sample was dissolved in 0.5 mL of deuterated chloroform (CDCl_3_). The scanning delay time interval was set at 1 s. Scanning times were set at 32. The frequency was 600 MHz.

### 2.11. Statistics

Independently replicated experiments were conducted with binding strengths expressed as mean ± standard deviation. Origin 8.5 and SPSS 26.0 were used to plot the data and analyze the significance (*p*-value < 0.05) among the data.

## 3. Results and Discussion

### 3.1. Effects of Antioxidant Types on Binding Strengths and Aging-Resistant Properties of ESO/DSO-Based PSAs

**Figure 1:**
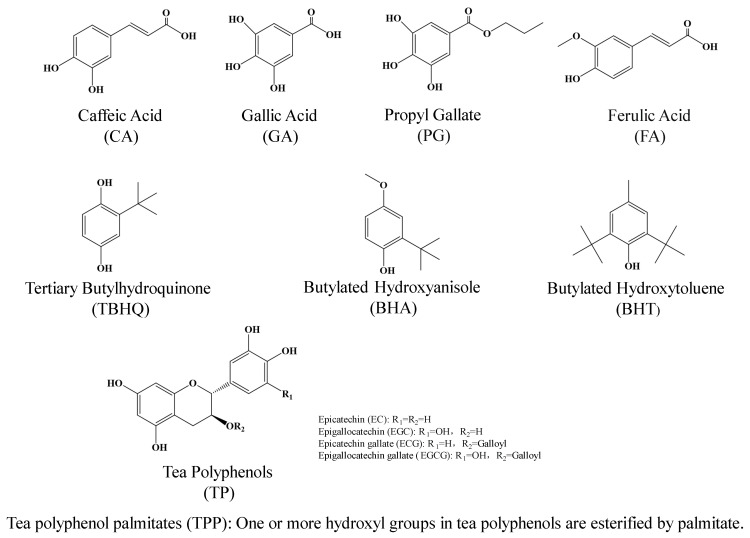
Chemical structures of antioxidants.

**Figure 2:**
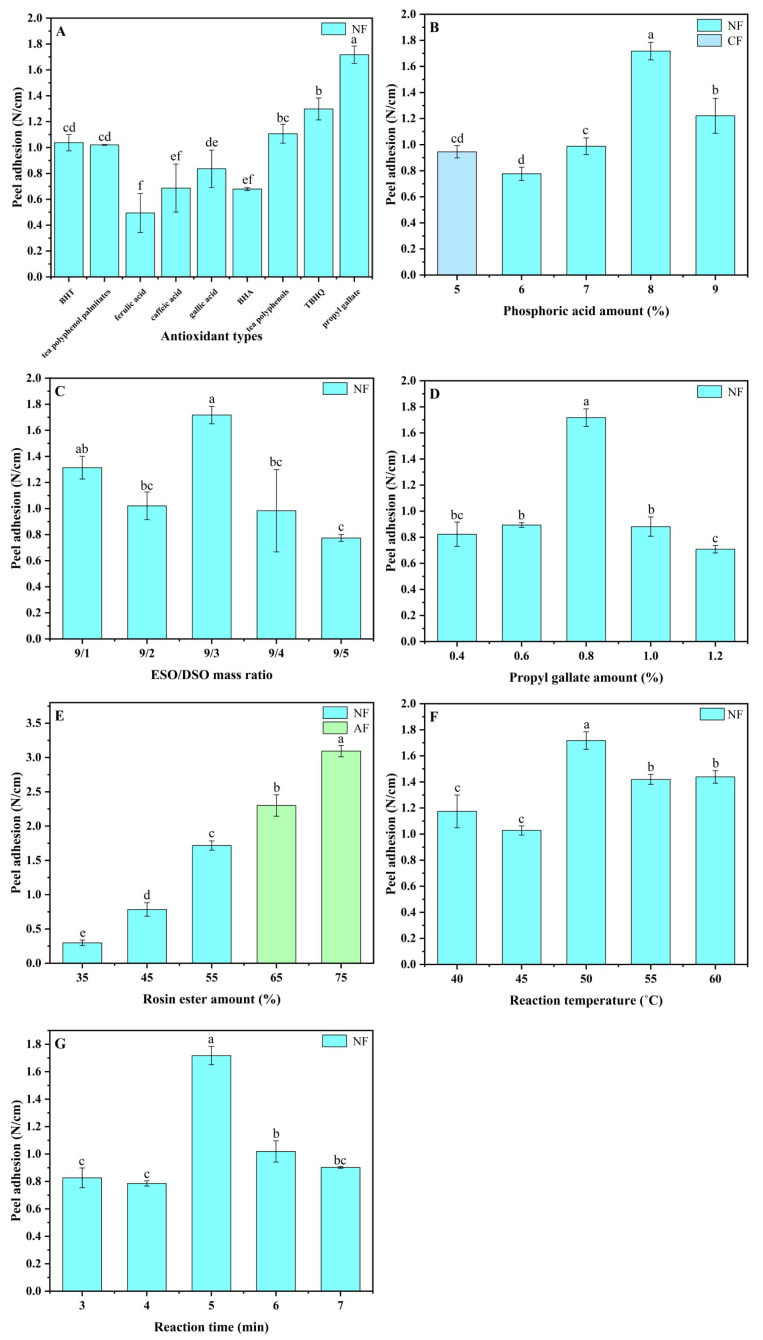
Effects of antioxidant types (**A**), phosphoric acid amount (**B**), ESO/DSO mass ratio (**C**), propyl gallate amount (**D**), rosin ester amount (**E**), reaction temperature (**F**), and reaction time (**G**) on peel adhesion of ESO/DSO-based PSAs (The different lowercase letters in each graph indicated the significant difference (*p* < 0.05)).

**Figure 3:**
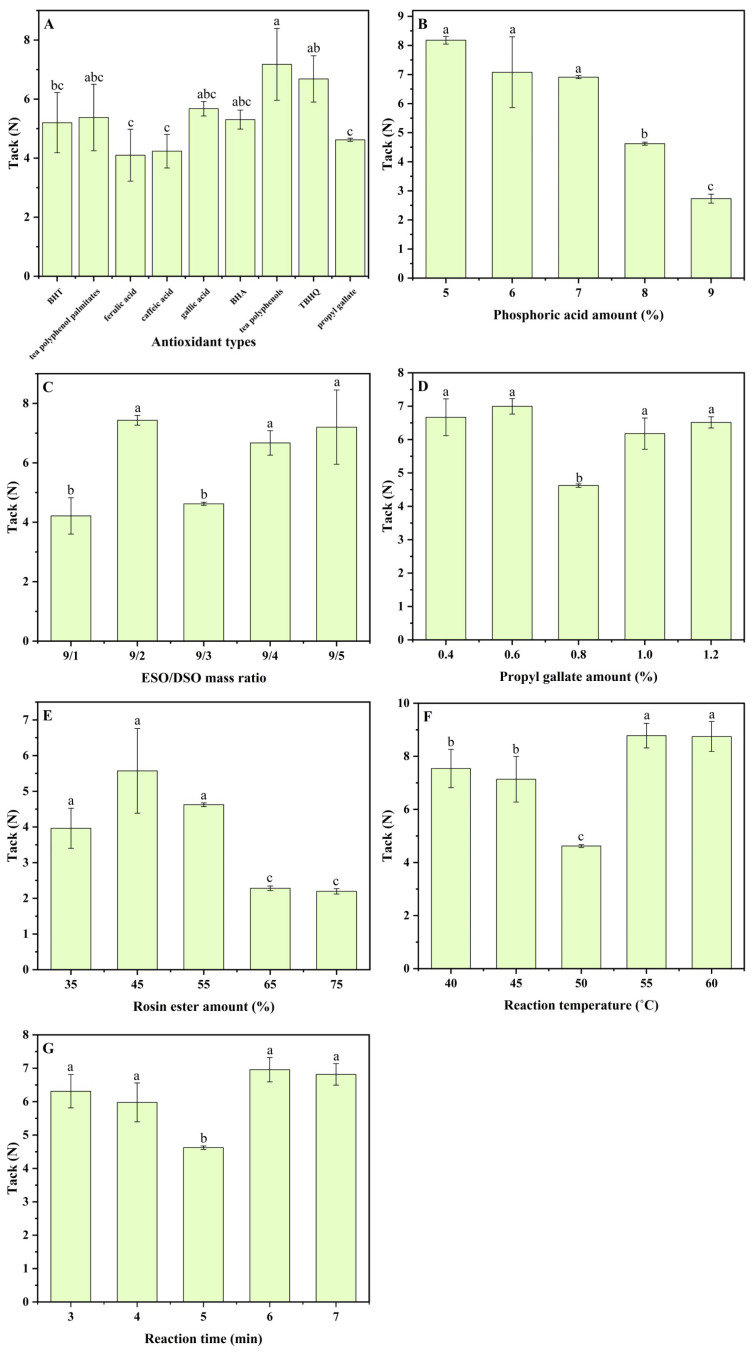
Effects of antioxidant types (**A**), phosphoric acid amount (**B**), ESO/DSO mass ratio (**C**), propyl gallate amount (**D**), rosin ester amount (**E**), reaction temperature (**F**), and reaction time (**G**) on tack of ESO/DSO-based PSAs (The different lowercase letters in each graph indicated the significant difference (*p* < 0.05)).

The nine common antioxidants (TP, TPP, BHA, BHT, TBHQ, PG, CA, FA, and GA) which this study attempted to graft onto ESO/DSO-based PSAs are shown in Figure 1. The structural differences among them determine that they could each perform differently in the resulting ESO/DSO-based PSAs. Thus, the effects of antioxidant types on the binding strengths and aging-resistant properties of ESO/DSO-based PSAs were studied (Figure 2A and Figure 3A and Table 1), with the levels of other factors being fixed (ESO/DSO mass ratio of 9/3, 8.0% PA, 55% RE, 0.8% antioxidant, 50 °C, and 5 min).

As expected, ESO/DSO-based PSAs had good softness. None of the ESO/DSO-based PSAs suffered failure, regardless of antioxidant types. The effects of antioxidant types on the peel adhesion of ESO/DSO-based PSAs followed the order (Figure 2A) PG > TBHQ > TP > BHT > TPP > GA > CA > BHA > FA. According to their chemical structures (Figure 1), FA (pKa1: 4.46, [20]), GA (pKa1: 4.26, [21]), and CA (pKa1: 4.38, [22]) all have phenolic hydroxyl and carboxylic groups, thus presenting acidity and leading to the excessive crosslinking of ESO/DSO-based PSAs. Among these three antioxidants, FA led ESO/DSO-based PSAs to have the worst binding performance (peel adhesion of 0.494 N/cm and tack of 4.10 N), which may be because FA has the smallest molar amount of polar functional groups under the same mass amount. Among the other antioxidants, BHA and BHT have the fewest hydroxyl groups and TP has the most hydroxyl groups. However, the steric hindrance of TP is more than that of TBHQ and PG [23]. Those factors comprehensively led the TP-grafted ESO/DSO-based PSA (1.107 N/cm) to have lower peel adhesion than the TBHQ- and PG-grafted ones (1.299 and 1.718 N/cm), but it led the TP- and TBHQ-grafted ESO/DSO-based PSAs (7.18 and 6.69 N) to have greater tack than the PG-grafted one (4.62 N). Compared to the TPP-grafted ESO/DSO-based PSA, the PG-grafted ESO/DSO-based PSA had higher peel adhesion (1.718 vs. 1.021 N/cm), which could be because the large molecular weight and steric hindrance of TPP led the resulting ESO/DSO-based PSA to have less cohesion strength. After aging, the TP- and PG-grafted ESO/DSO-based PSAs did not suffer failure, the TBHQ-grafted ESO/DSO-based PSA suffered adhesive failure (AF), and the remaining six suffered cohesive failure (CF) (Table 1). The peel adhesion residues of the TP- and PG-grafted ESO/DSO-based PSAs were 4.00 and 12.16%, respectively. After comprehensive consideration, PG was selected as the most suitable antioxidant.

### 3.2. Effects of PA Amount on Binding Strengths and Aging-Resistant Properties of ESO/DSO-Based PSAs

The effects of the PA amount on the binding strengths and aging-resistant properties of ESO/DSO-based PSAs were studied (Figure 2B and Figure 3B and Table 1), with the levels of other factors being fixed (ESO/DSO mass ratio of 9/3, 55% RE, 0.8% PG, 50 °C, and 5 min).

With 5% PA, the PSA suffered CF due to low cohesion strength and small molecular weight (Figure 2B). When PA increased from 6 to 9%, peel adhesion showed an initially increasing and then a decreasing trend with the maximum (1.718 N/cm) at 8% PA (Figure 2B), while tack decreased continuously from 7.08 to 2.73 N (Figure 3B). The same trend was observed by Liu et al. [24], in whose study butyl acrylate and methyl methacrylate were copolymerized to prepare PSAs. With the increase in PA from 6 to 8%, the cohesion strength and creep resistance of PSA were enhanced, leading peel adhesion to gradually become higher. With the further increase of PA from 8 to 9%, serious entanglement among polymer molecular chains caused intermolecularly excessive crosslinking, reducing the peel adhesion. After aging, the ESO/DSO-based PSAs with 7% and 9% PA suffered AF and CF, respectively, while the ESO/DSO-based PSAs with 6% and 8% PA did not suffer failure (Table 1). However, the ESO/DSO-based PSA with 8% PA had a much lower peel adhesion residue (12.16%) than that with 6% PA (342.39%). After comprehensive consideration, therefore, 8% PA was chosen as the most suitable amount.

### 3.3. Effects of ESO/DSO Mass Ratio on Binding Strengths and Aging-Resistant Properties of ESO/DSO-Based PSAs

The effects of the ESO/DSO mass ratio on the binding strengths and aging-resistant properties of ESO/DSO-based PSAs were studied (Figure 2C and Figure 3C and Table 1), with the levels of other factors being fixed (8.0% PA, 55% RE, 0.8% PG, 50 °C, and 5 min).

When the mass ratio of ESO/DSO was at 9/1–9/2, tack increased from 4.22 to 7.43 N with the increase in DSO. As a small-molecular-weight substance, DSO has certain intermolecular resistance in the PSA system due to its high viscosity, reducing peel adhesion from 1.314 to 1.021 N/cm. As the mass ratio of ESO/DSO further increased to 9/3, more DSO participated in crosslinking rather than presenting in the free form, increasing peel adhesion to 1.718 N/cm but reducing tack to 4.62 N. When the mass ratio of ESO/DSO was at 9/3–9/5, the solution became very sticky with the increase in DSO, and the chances of intermolecular-collision crosslinking were reduced, lowering the degree of cohesion crosslinking and decreasing the peel adhesion. Excessive DSO presented in the polymer system in the free form, leading the amount of polar functional groups to increase and thus increasing tack. After aging, only the ESO/DSO-based PSA with the ESO/DSO mass ratio of 9/3 did not suffer failure (Table 1). This indicates that a too high or too low ESO/DSO mass ratio was not unfavorable. Thus, 9/3 was chosen as the most suitable mass ratio of ESO/DSO.

### 3.4. Effects of PG Amount on Binding Strengths and Aging-Resistant Properties of ESO/DSO-Based PSAs

The effects of the PG amount on the binding strengths and aging-resistant properties of ESO/DSO-based PSAs were studied (Figure 2D and Figure 3D and Table 1), with the levels of other factors being fixed (ESO/DSO mass ratio of 9/3, 8.0% PA, 55% RE, 50 °C, and 5 min).

With the increase in the PG amount, peel adhesion increased initially and then decreased, while tack did the opposite. With the increase in the PG amount from 0.4 to 0.6%, peel adhesion and tack increased from 0.823 to 0.894 N/cm and 6.67 to 7.00 N, respectively, because of the increased number of hydroxyl groups available for cohesion crosslinking and wettability improving. When the PG amount was 0.8%, cohesion strength reached the maximum, with a lower number of free hydroxyl groups, resulting in peel adhesion presenting the maximum but tack presenting the minimum. When the PG amount exceeded 0.8%, the adverse effects from the steric hindrance of PG significantly hindered the effective crosslinking of polymers, reducing the peel adhesion, while tack increased due to the presence of excessive free hydroxyl groups. After aging, the ESO/DSO-based PSA with 0.8% PG did not suffer failure, while others suffered CF, AF, or mixed failure (MF) (Table 1). Therefore, 0.8% was chosen as the most suitable amount of PG.

### 3.5. Effects of RE Amount on Binding Strengths and Aging-Resistant Properties of ESO/DSO-Based PSAs

The effects of the RE amount on the binding strengths and aging-resistant properties of ESO/DSO-based PSAs were studied (Figure 2E and Figure 3E and Table 1), with the levels of other factors being fixed (ESO/DSO mass ratio of 9/3, 8.0% PA, 0.8% PG, 50 °C, and 5 min).

With the increase of the RE amount from 35 to 75%, peel adhesion increased from 0.297 to 3.094 N/cm because of the increased cohesion strength. However, when the RE amount was more than 65%, the creep resistance of the PSA became worse, but the rigidity increased, causing AF. With the increase of the RE amount from 35 to 45%, the wettability of the PSA increased due to the introduction of more polar functional groups, enhancing the tack. When the RE amount was at 45–65%, the serious entanglement among polymer molecular chains led the RE to be wrapped in the network structures of the PSA and the number of polar functional groups exposed at the surface of the PSA to be reduced, reducing the tack. After aging, the peel adhesion residues of the ESO/DSO-based PSAs followed the order 35% RE > 45% RE > 55% RE (Table 1). In addition, the ESO/DSO-based PSAs with 35% and 45% RE suffered CF, while the ESO/DSO-based PSA with 55% RE did not suffer failure. Therefore, 55% was chosen as the most suitable amount of RE.

### 3.6. Effects of Reaction Temperature on Binding Strengths and Aging-Resistant Properties of ESO/DSO-Based PSAs

The effects of the reaction temperature on the binding strengths and aging-resistant properties of ESO/DSO-based PSAs were studied (Figure 2F and Figure 3F and Table 1), with the levels of other factors being fixed (ESO/DSO mass ratio of 9/3, 8.0% PA, 55% RE, 0.8% PG, and 5 min).

When the reaction temperature was 40 °C, the insufficient cohesion strength caused a shadow to be present on the steel test plate, but without residual adhesives when peeling. When the reaction temperature was 45–50 °C, the chances of molecular collision became greater and the degree of crosslinking became larger, enhancing the peel adhesion but reducing the tack because of the reduced number of polar functional groups. When the reaction temperature reached 50–55 °C, molecular collision occurred more easily and molecular softness became worse, reducing the peel adhesion to 1.420 N/cm. The accelerated molecular collision led epoxy groups to be rapidly crosslinked with the adjacent functional groups after ring opening, the network structures of the PSA to be loose, and more polar functional groups to be exposed, enhancing the tack to 8.78 N. When the reaction temperature was 55–60 °C, the bonding strengths of the PSAs did not change significantly. After aging, the peel adhesion residues of the ESO/DSO-based PSAs showed an initially decreasing and then an increasing trend with the increased reaction temperatures (Table 1). Only the ESO/DSO-based PSA with a reaction temperature of 50 °C did not suffer failure. Therefore, 50 °C was chosen as the most suitable reaction temperature.

### 3.7. Effects of Reaction Time on Binding Strengths and Aging-Resistant Properties of ESO/DSO-Based PSAs

The effects of the reaction temperature on the binding strengths and aging-resistant properties of ESO/DSO-based PSAs were studied (Figure 2G and Figure 3G and Table 1), with the levels of other factors being fixed (ESO/DSO mass ratio of 9/3, 8.0% PA, 55% RE, 0.8% PG, and 50 °C).

With the extension of the reaction time, peel adhesion increased initially and then decreased, while tack did the opposite. When the reaction time was within 3 min, the insufficient cohesion strength caused a shadow to be presented on the steel test plate when peeling. When the reaction time was 4–5 min, the increased degree of crosslinking enhanced the peeling adhesion but reduced the tack because of the reduced number of polar functional groups caused by polyesterification and polyetherification. With the further extension of the reaction time to 6 min, excessive crosslinking decreased the peel adhesion to 1.019 N/cm, while the tack increased to 6.96 N because the polar functional groups generated from the ring opening of epoxy groups at the late stage of reaction had to be exposed outside the network structures after the network structures of the PSA were constructed at the early and middle stages of the reaction. When the reaction time was 6–7 min, the bonding strengths of the PSAs did not change significantly. After aging, the ESO/DSO-based PSA with a reaction time of 5 min did not suffer failure (Table 1), while others suffered AF. Therefore, 5 min was chosen as the most suitable reaction time.

### 3.8. Comparison of Binding Strengths and Aging-Resistant Properties of ESO/DSO-Based PSAs with and without PG Grafting

The binding strengths and aging-resistant properties of ESO/DSO-based PSAs with and without PG grafting were compared under the above optimized conditions, giving the results shown in Table 2.

Before aging, PG grafting largely enhanced the shear adhesion, peel adhesion and tack: from 13.88 to >99 h, 0.879 to 1.718 N/cm, and 3.59 to 4.62 N, respectively. This is because PG not only improved the degree of crosslinking polymerization of the ESO/DSO-based PSA, but also increased the number of polar functional groups in the polymer, enhancing the binding strengths of the ESO/DSO-based PSA. After aging, the shear adhesion of both the control and the PG-grafted ESO/DSO-based PSAs was greater than 99 h, and peel adhesion increased but tack decreased because of the occurrence of further crosslinking polymerization under the action of dry-heat aging. The peel adhesion residue of the PG-grafted ESO/DSO-based PSA (12.16%) was much lower than that of the control (484.07%), indicating that PG greatly improved the aging-resistant property of the ESO/DSO-based PSA.

### 3.9. Thermal Properties

**Figure 4:**
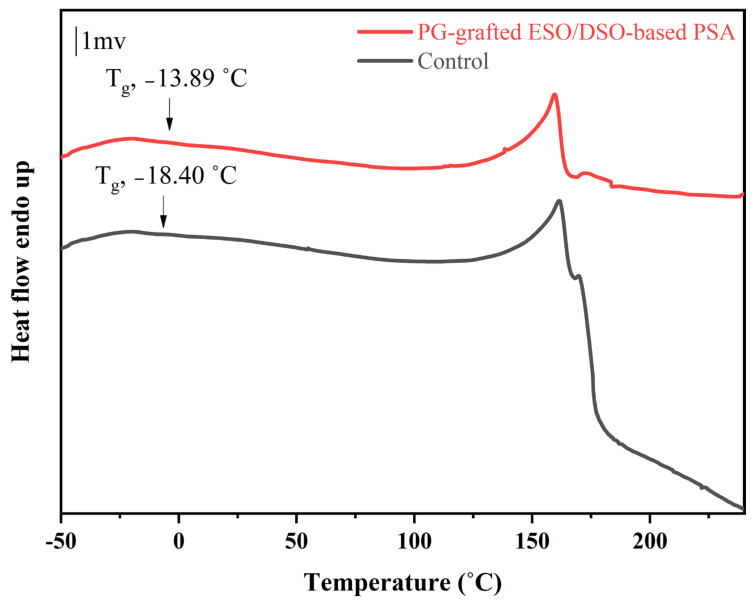
Differential scanning calorimetry thermograms of ESO/DSO-based PSAs with and without propyl gallate grafting [T_g_, glass transition temperature].

**Figure 5:**
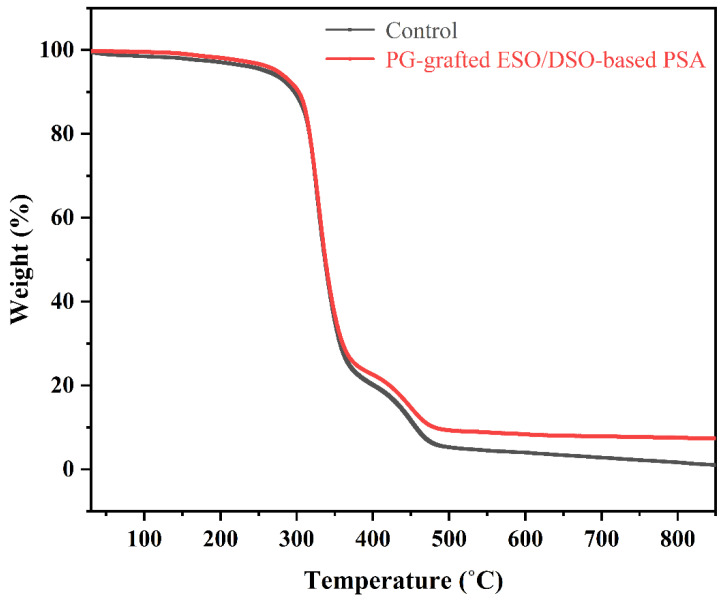
Thermogravimetric thermograms of ESO/DSO-based PSAs with and without propyl gallate grafting.

The thermal properties of ESO/DSO-based PSAs with and without PG grafting are shown in Figure 4 and Figure 5 and Table 3.

Compared to the control, the PG-grafted ESO/DSO-based PSA had higher T_g_ (−13.89 vs. −18.40 °C, Figure 4) and larger initial and maximum decomposition temperatures (303 vs. 297 °C, 481 vs. 456 °C, Table 3), but a similar fastest decomposition temperature (328 vs. 327 °C). This is attributed to the introduction of PG and the enhanced degree of crosslinking.

### 3.10. FTIR Analysis

**Figure 6:**
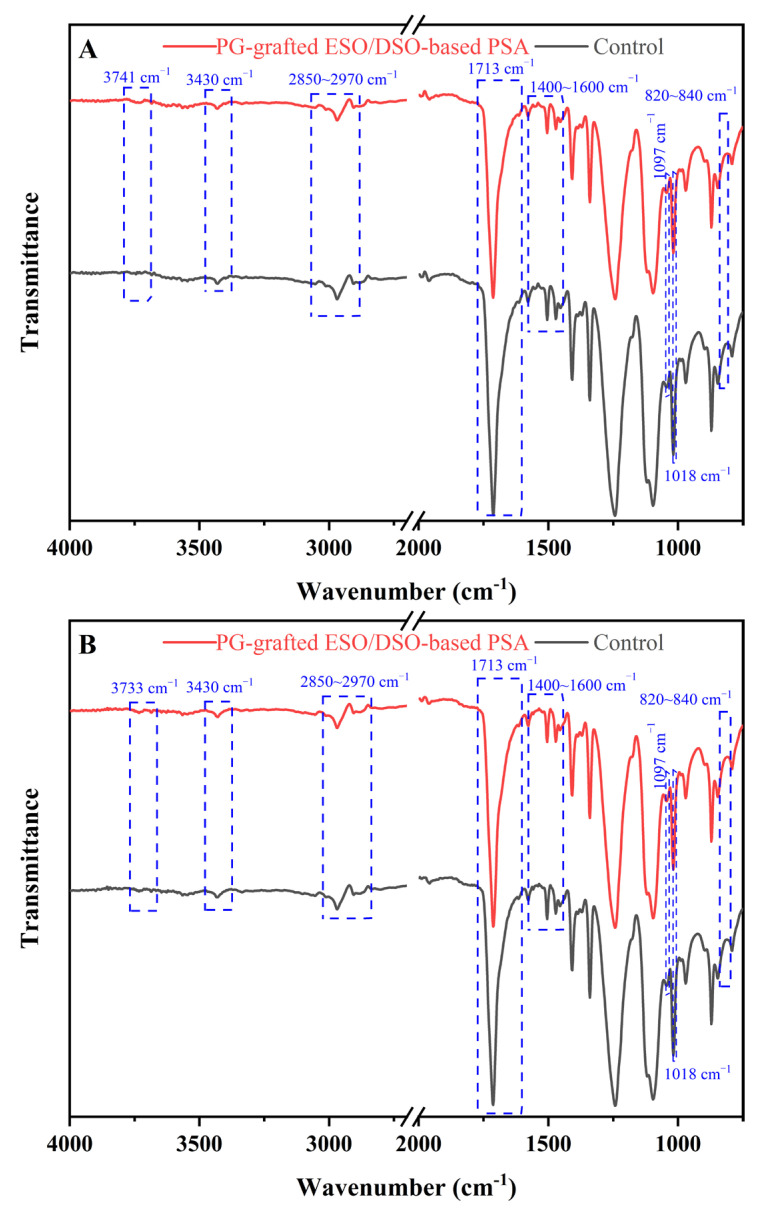
Fourier-transform infrared spectra of ESO/DSO-based PSAs with and without propyl gallate grafting before (**A**) and after (**B**) aging.

The FTIR analysis of the ESO/DSO-based PSAs with and without PG grafting before and after aging are shown in Figure 6.

Before aging, the peaks of the epoxy groups (820–840 cm^−1^) weakened and the peak of the free hydroxyl groups (3741 cm^−1^) enhanced (Figure 6A), which indicates the ring opening of the epoxy groups [25]. The peak of the ester bonds of ESO and DSO was at 1713 cm^−1^ [26]. The peak of the ether bonds at 1018 cm^−1^ indicates that polyetherification occurred accompanying the ring opening of the epoxy groups [27]. The peaks of the phosphate ester bonds at 1097 cm^−1^ indicate that PA participated in the crosslinking polymerization [27]. The vibrating peaks of the benzene ring of PG were at 1400–1600 cm^−1^, and the vibrating peaks of the methyl and methylene groups of ESO and DSO were at 1380–1470 cm^−1^, leading them to overlap [26]. The vibrating peaks of methyl and methylene of ESO and DSO as well as the hydroxyl groups on the free carboxyl groups overlapped at 2850–2970 cm^−1^ [28]. Compared with the control, the PG-grafted ESO/DSO-based PSA showed an enhanced free hydroxyl peak at 3741 cm^−1^ due to the introduction of phenolic hydroxyl groups of PGs. The peak representing the associated hydroxyl groups appeared at 3430 cm^−1^, suggesting that partially free hydroxyl groups were connected with hydrogen bonds. As shown in Figure 6B, the peak at 3733 cm^−1^ became sharp after aging, which is because dry-heat aging led free hydroxyl groups to be further polymerized, aggravating the structural asymmetry of the PSA polymer.

### 3.11. ^1^H NMR Analysis

**Figure 7:**
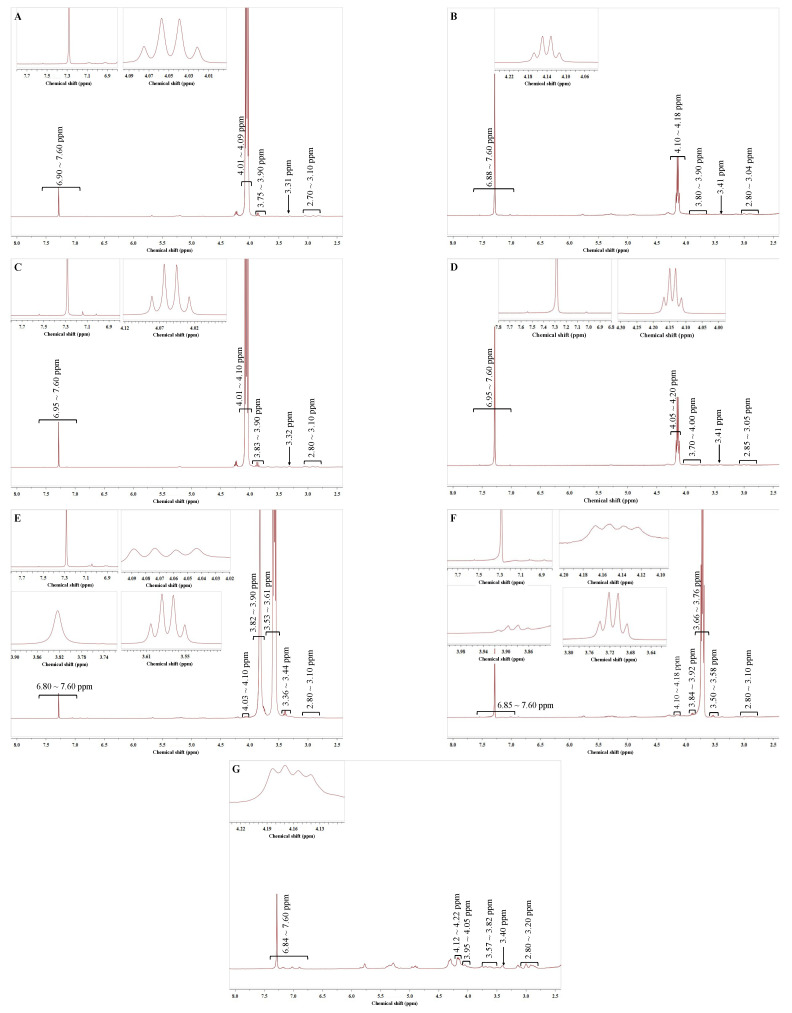
^1^H NMR spectra of ESO/DSO-based PSAs with and without propyl gallate grafting and/or addition of rosin ester. (**A**): ethyl acetate solution that soaked ESO/DSO-based PSA (without propyl gallate grafting but with addition of rosin ester); (**B**): ESO/DSO-based PSA (without propyl gallate grafting but with addition of rosin ester) residue after soaking with ethyl acetate; (**C**): ethyl acetate solution that soaked PG-grafted ESO/DSO-based PSA (without addition of rosin ester); (**D**): PG-grafted ESO/DSO-based PSA (without addition of rosin ester) residue after soaking with ethyl acetate; (**E**): anhydrous ethanol solution that soaked PG-grafted ESO/DSO-based PSA (with addition of rosin ester); (**F**): PG-grafted ESO/DSO-based PSA (with addition of rosin ester) residue after soaking with anhydrous ethanol; (**G**): PG-grafted ESO/DSO-based PSA (with addition of rosin ester).

The ^1^H NMR spectra of ESO/DSO-based PSAs with and without PG grafting and/or the addition of RE are shown in Figure 7.

The ESO/DSO-based PSA without PG grafting but with the addition of RE had the peaks of benzene ring at δ 6.88–7.60 ppm [29] both in its acetate ethyl soaking solution and in its solid residue after acetate ethyl soaking (Figure 7A,B), indicating that RE was partially free and partially embedded in the network structures of the PSA. Similarly, the ESO/DSO-based PSA with PG grafting but without the addition of RE also had the peaks of benzene ring at δ 6.95–7.60 ppm both in its acetate ethyl soaking solution and in its solid residue after acetate ethyl soaking (Figure 7C,D), indicating that PG was also partially free and partially crosslinked in the network structures of the PSA. The ESO/DSO-based PSA with PG grafting and the addition of RE had the peaks of mono- or oligohydroxyl groups at δ 3.82–3.92 ppm [30] and the peaks of polyhydroxyl groups at δ 3.36–3.58 ppm [31], indicating that PG was also partially free and partially crosslinked in the network structures of the PSA. Combining the analysis from Figure 7A–F, the peaks at δ 6.84–7.60 ppm in Figure 7G were due to the benzene ring structures of both PG and RE. The quadruple peaks at δ 4.12–4.22 ppm were due to PA and its esters [32]. The peaks at δ 3.95–4.05, 3.57–3.82, 3.40, and 2.80–3.20 ppm were due to oligo-hydroxyl groups, ether bonds [33], polyhydroxyl groups, and hydrogen of epoxy groups [34], respectively.

## 4. Conclusions

Among the nine common antioxidants, PG comprehensively presented the best performance in effectively enhancing the binding strengths and aging-resistant properties of ESO/DSO-based PSAs. The optimal conditions were ESO/DSO mass ratio of 9/3, 0.8% PG, 55% RE, and 8% PA at 50 °C for 5 min. Compared with the control, PG grafting increased peel adhesion, tack, and shear adhesion by 2.0, 1.3, and >7.1 times, respectively, and enhanced the aging-resistant property as peel adhesion residue reduced by 97.5%. PG, RE, PA, and DSO were partially crosslinked in the PSA system with the rest being free in the network structures. Antioxidant grafting is a great pattern for improving the binding strengths and aging-resistant properties of vegetable-oils-based PSAs, which could improve the competitiveness of vegetable-oils-based PSAs in the whole PSA market.

## Figures and Tables

**Table 1:** Effects of antioxidant types, phosphoric acid amount, ESO/DSO mass ratio, propyl gallate amount, rosin ester amount, reaction temperature, and reaction time on the peel adhesion residue of ESO/DSO-based PSAs (only the levels for each factor under which conditions ESO/DSO-based PSAs did not suffer failure were used for the investigation of peel adhesion residue).

Factors	Peel Adhesion before Aging (N/cm)	Peel Adhesion after Aging (N/cm)	Failure Form after Aging	Peel Adhesion Residue (%)
Antioxidant types				
BHT	1.038 ± 0.06 ^cd^	5.463 ± 1.03 ^a^	CF	426.15
Tea polyphenol palmitates	1.021 ± 0.00 ^cd^	4.974 ± 0.01 ^a^	CF	369.62
Ferulic acid	0.494 ± 0.15 ^f^	1.401 ± 0.04 ^cd^	CF	183.44
Caffeic acid	0.687 ± 0.19 ^ef^	1.381 ± 0.41 ^cd^	CF	101.17
Gallic acid	0.836 ± 0.14 ^de^	0.310 ± 0.02 ^d^	CF	62.97
BHA	0.680 ± 0.01 ^ef^	0.622 ± 0.13 ^d^	CF	8.58
Tea polyphenols	1.107 ± 0.07 ^bc^	1.063 ± 0.32 ^cd^	NF	4.00
TBHQ	1.299 ± 0.08 ^b^	2.557 ± 0.71 ^b^	AF	96.91
Propyl gallate	1.718 ± 0.07 ^a^	1.927 ± 0.24 ^bc^	NF	12.16
Phosphoric acid amount (%)				
6.0	0.777 ± 0.05 ^c^	3.437 ± 0.10 ^b^	NF	342.39
7.0	0.988 ± 0.06 ^bc^	4.511 ± 0.13 ^a^	AF	356.54
8.0	1.718 ± 0.07 ^a^	1.927 ± 0.24 ^c^	NF	12.16
9.0	1.222 ± 0.13 ^b^	5.388 ± 0.67 ^a^	CF	341.01
ESO/DSO mass ratio				
9/1	1.314 ± 0.09 ^ab^	4.216 ± 0.24 ^b^	CF	220.85
9/2	1.021 ± 0.11 ^bc^	8.576 ± 1.00 ^a^	CF	739.86
9/3	1.718 ±0.07 ^a^	1.927 ± 0.24 ^c^	NF	12.16
9/4	0.983 ± 0.32 ^bc^	4.992 ± 0.35 ^b^	CF	407.61
9/5	0.774 ± 0.03 ^c^	5.066 ± 0.66 ^b^	CF	554.28
Propyl gallate amount (%)				
0.4	0.823 ± 0.09 ^bc^	7.216 ± 0.15 ^a^	CF	776.61
0.6	0.894 ± 0.02 ^b^	6.147 ± 0.00 ^ab^	CF	587.67
0.8	1.718 ± 0.07 ^a^	1.927 ± 0.24 ^c^	NF	12.16
1.0	0.882 ± 0.07 ^b^	6.824 ± 0.15 ^ab^	MF	673.82
1.2	0.709 ± 0.03 ^c^	5.377 ± 1.24 ^b^	AF	658.80
Rosin ester amount (%)				
35	0.297 ± 0.04 ^c^	4.633 ± 0.72 ^a^	CF	1458.30
45	0.784 ± 0.10 ^b^	3.969 ± 0.05 ^a^	CF	406.08
55	1.718 ± 0.07 ^a^	1.927 ± 0.24 ^b^	NF	12.16
Reaction temperature (°C)				
40	1.175 ± 0.13 ^c^	9.174 ± 0.78 ^a^	AF	681.00
45	1.028 ± 0.04 ^c^	3.404 ± 0.29 ^b^	AF	231.16
50	1.718 ± 0.07 ^a^	1.927 ± 0.24 ^b^	NF	12.16
55	1.420 ± 0.04 ^b^	8.562 ± 1.05 ^a^	AF	503.05
60	1.439 ± 0.05 ^b^	9.121 ± 0.21 ^a^	AF	533.99
Reaction time (min)				
3	0.826 ± 0.07 ^c^	8.188 ± 0.87 ^a^	AF	891.10
4	0.786 ± 0.02 ^c^	8.395 ± 0.77 ^a^	AF	968.48
5	1.718 ± 0.07 ^a^	1.927 ± 0.24 ^b^	NF	12.16
6	1.019 ± 0.08 ^b^	7.786 ± 0.68 ^a^	AF	664.30
7	0.902 ± 0.01 ^bc^	8.662 ± 0.32 ^a^	AF	859.90

AF: adhesive failure; CF: cohesive failure; MF: mixed failure. The different lowercase letters in each column under each factor indicated the significant difference (*p* < 0.05).

**Table 2:** Comparison of binding strengths and aging-resistant properties of ESO/DSO-based PSAs with and without propyl gallate grafting before and after aging.

ESO/DSO-Based PSAs	Before Aging			After Aging			Peel Adhesion Residue (%)
	Peel Adhesion (N/cm)	Tack (N)	Shear Adhesion (h)	Peel Adhesion (N/cm)	Tack (N)	Shear Adhesion (h)	
Control	0.879 ± 0.18 ^b^	3.59 ± 0.38 ^b^	13.88 ± 3.93 ^b^	5.134 ± 0.58 ^a^	1.14 ± 0.22 ^b^	>99	484.07
Propyl gallate-grafted	1.718 ± 0.07 ^a^	4.62 ± 0.05 ^a^	>99 ^a^	1.927 ± 0.24 ^b^	2.28 ± 0.06 ^a^	>99	12.16

The different lowercase letters in each column indicate the significant difference (*p* < 0.05).

**Table 3:** Thermal decomposition temperatures of ESO/DSO-based PSAs with and without propyl gallate grafting.

ESO/DSO-Based PSAs	T_d,10%_ (°C)	T_d,90%_ (°C)	T_d,max_ (°C)
Control	297	456	327
Propyl gallate-grafted	303	481	328

T_d,10%_: temperature at which weight loss was up to 10%; T_d,90%_: temperature at which weight loss was up to 90%; T_d,max_: temperature at which maximum weight-loss rate occurred.

## Data Availability

The data used for the research described in this manuscript are available upon request.
